# Effects of endocrine disruptors on fetal testis development, male puberty, and transition age

**DOI:** 10.1007/s12020-020-02436-9

**Published:** 2020-08-05

**Authors:** Francesco Cargnelutti, Andrea Di Nisio, Francesco Pallotti, Iva Sabovic, Matteo Spaziani, Maria Grazia Tarsitano, Donatella Paoli, Carlo Foresta

**Affiliations:** 1 Laboratory of Seminology-Sperm Bank “Loredana Gandini”, Department of Experimental Medicine, “Sapienza” University of Rome, Viale del Policlinico 155, 00161 Rome, Italy; 2 Department of Medicine, Operative Unit of Andrology and Medicine of Human Reproduction, University of Padova, Via Giustiniani, 2, 35128 Padua, Italy; 3 Department of Experimental Medicine, “Sapienza” University of Rome, Viale del Policlinico 155, 00161 Rome, Italy

**Keywords:** Endocrine disruptors, Male reproductive health, Testicular function, Testicular cancer

## Abstract

**Purpose:**

Endocrine disruptors (EDs) are exogenous substances able to impair endocrine system; consequently, they may cause numerous adverse effects. Over the last years, particular focus has been given to their harmful effects on reproductive system, but very little is known, especially in males. The aim of this review is to discuss the detrimental effects of EDs exposure on fetal testis development, male puberty, and transition age.

**Methods:**

A search for the existing literature focusing on the impact of EDs on fetal testis development, male puberty, andrological parameters (anogenital distance, penile length, and testicular volume), and testicular cancer with particular regard to pubertal age provided the most current information available for this review. Human evidence-based reports were given priority over animal and in vitro experimental results. Given the paucity of available articles on this subject, all resources were given careful consideration.

**Results:**

Information about the consequences associated with EDs exposure in the current literature is limited and often conflicting, due to the scarcity of human studies and their heterogeneity.

**Conclusions:**

We conclude that current evidence does not clarify the impact of EDs on human male reproductive health, although severe harmful effects had been reported in animals. Despite controversial results, overall conclusion points toward a positive association between exposure to EDs and reproductive system damage. Further long-term studies performed on wide number of subjects are necessary in order to identify damaging compounds and remove them from the environment.

## Introduction

According to the WHO/IPCS 2002 definition, “an Endocrine Disruptor (ED) is an exogenous substance or mixture that alters functions of the endocrine system and consequently causes adverse effects in an intact organism, or its progeny.” Over the past two decades, public health has focused on the identification of environmental EDs that are able to adversely affect hormonal function [1]. EDs mimic naturally occurring hormones like estrogens and androgens and exert their toxicity by interfering with the normal hormonal homeostatic mechanisms that promote growth and development of tissues. EDs usually interfere with the hormonal binding to the corresponding receptor, notably the androgen receptor (AR) or the estrogen receptor. Subsequently, EDs can trigger two types of response: agonistic and/or antagonistic effect. In addition, recent discoveries in molecular biology confirmed a possible interference by several compounds with the cell cycle, the apoptotic mechanisms, and the epigenetic regulation [2]. Epigenetic changes are able to modify activation and expression of genes, though not altering the genetic code sequence. Changes in DNA methylation, histone modifications, and noncoding RNAs are involved. Recent studies reported a transmission of epigenetic shifts from father to child, suggesting a transgenerational inheritance [3, 4]. Therefore, EDs may have negative effects not only in exposed individuals, but also in their offspring and in future generations. Main EDs characteristics are summarized in Table 1. In addition to exposure by direct contact with these materials, EDs are also released into the environment. Therefore, exposure may occur through food and water consumption, inhalation, or dermal contact. During fetal and neonatal life it could also occur through placenta and breast feeding. As a consequence, EDs may affect human pre and postnatal development. In fact, infants can be affected already at prenatal level due to maternal exposure to EDs [5]. The effects of EDs on the male reproductive system are usually attributed to the interactions of these chemicals with the normal production and/or function of steroid hormones that are responsible for the masculinization of the Wolffian ducts [6]. In males, reproductive disorders associated with impaired fetal testis development or function vary in both phenotype and time of manifestation. Often gathered under the “testicular dysgenesis syndrome” (TDS) hypothesis [7], these male disorders range from hypospadias and cryptorchidism in infants [8–11] to low testosterone levels, infertility, and testicular cancer (TC) in adult men [12–14]. TDS has been correlated with environmental factors during fetal life [7].

**Table 1:** Classification, properties, and effects on male reproductive system of most common EDs

EDs	Group	Chemical structure	Main sources	Half-life	Main effect
Phthalates	Plasticizers		Polyvinyl chloride (PVC) products, toys, medical devices, cosmetics, and personal care products	About 12 h	Antiandrogenic
Bisphenol A (BPA)	Bisphenols		Polycarbonate plastics, epoxy resins, plastic toys, and bottles	4–5 h	Estrogenic
Dichlorodiphenyldichloroethylene (DDE)	Organochlorides		Pesticides (and contaminated water, soil products, fish)	About 8 years	Antiandrogenic, estrogenic
Polychlorinated biphenyls (PCBs)	Organochlorides		Pesticides, flame retardants (and contaminated water, soil products, fish)	Up to 16 years	Variable (estrogenic, antiestrogenic or antiandrogenic)
Per- and poly-fluoroalkyl substances (PFAS)	Fluorosurfactant		Commercial household products (e.g., stain- and water-repellent fabrics), electronics manufacturing (and contaminated water, soil products, fish)	Up to 10 years	Antiandrogenic, antiestrogenic

The aim of this review is to discuss the detrimental effects of EDs exposure on fetal testis development, male puberty, and transition age, the latter defined as age 18–25 years. This definition is consistent with the recent discoveries in the field of neurophysiology, in fact a maturation of prefrontal cortex until 25 years old has been pointed out [15, 16]. This review aims to provide an overview of animal, in vitro, and human studies, though human evidence-based reports were given priority. In particular, andrological parameters of male health such as anogenital distance (AGD), penile length, and testicular volume (TV) were investigated.

## Effects of EDs on testis development

Testis development during fetal life is crucial for male reproductive function in adulthood [17]. Indeed, fetal period is critical for the regular development of the testis and is known as a period of high sensitivity to many EDs [18]. Both functions of testis (spermatogenesis and steroidogenesis) are set up early during fetal life [19]. Primordial gonads appear between the 4th and the 6th week post fertilization. They are rapidly colonized by primordial germ cells that migrate from extra-embryonic areas [20]. Six weeks post fertilization, the differentiation of the testis is due to Sertoli cells surrounding testicular cords [21]. Sertoli cells are central for germ cell development [22] and Leydig cells differentiation [23]. Indeed, at 6th week post fertilization, fetal Leydig cells start producing testosterone and insulin-like factor 3 (INSL3). Both hormones are involved in testicular descent [24]. Furthermore, testosterone is crucial for fetal masculinization. Therefore, perturbations to the function of fetal Leydig cells can predispose to the development of male reproductive disorders including TDS and other disorders of sex development [25].

In the last years, literature focused on possible association between EDs and testis development. Several experimental animal studies were conducted to investigate a possible correlation, especially in mouse models. Di-2-ethylhexyl phthalate (DEHP) and di-n-butyl phthalate (DBP) are the most abundant phthalates which, after ingestion, are hydrolyzed into the active monoesters monoethylhexyl phthalate (MEHP) and mono-n-butyl phthalate (MBP), respectively. In rodents, the effects of DEHP and DBP exposure in utero have been largely described. Numerous studies reported a disruption of normal fetal testis development and the subsequent development of male reproductive disorders [17, 26]. It is interesting to note that in mice phthalates can induce a positive effect on testosterone secretion by the cultured fetal testis [27, 28]. Surprisingly, such effect was not observed in the human testis. In this regard, both organ cultures and xenograft experiments revealed no marked change in testosterone production [29–31], suggesting that exposure to environmental levels of DBP and DEHP is unlikely to result in effects on fetal testosterone production in humans. Notably, although no effects of phthalate exposure have been demonstrated in human fetal testes, antiandrogenic effects occur in adult human testis following in vitro culture. The different effect of exposure could depend on the developmental stage of the testis [32]. However, experimental studies in rodents and human fetal tissues are consistent regarding germ cells, showing a reduction in the gonocyte number following phthalate exposure [30, 33]. Concerning other EDs, inconsistent results on testosterone production and germ cell development have been identified in animal studies investigating the effects of BPA exposure on fetal testis development [34, 35]. In the same way, inconsistent results on association between BPA and clinical indicators of reduced fetal testosterone (cryptorchidism and hypospadias) have been reported in epidemiological studies [36, 37]. For human testicular tissue experiments, in vitro studies indicate the potential for BPA to reduce testosterone production in the fetal testicle, whereas xenotransplantation studies failed to demonstrate similar effects [34, 35]. In addition, in vivo human BPA exposure might be below the concentrations used for experimental studies involving animal or human tissues [38]. Regarding other EDs, PCBs have been associated with abnormal urogenital development in animal models [39, 40]. In particular, lactational exposure seems to affect histology of rat testis in both prepuberal and puberal F1 progeny [41]. In rats, perfluorooctanoic acid (PFOA) does not seem to affect fetal Sertoli cells but may increase tendency of apoptosis in fetal Leydig cells [42]. This damage seems to affect both proliferation and differentiation of stem Leydig cells or their progeny [43]. Regarding perfluorooctanesulfonic acid (PFOS), it seems to damage Sertoli cells by perturbing actin cytoskeleton in primary cultures of rodent and human [44, 45] and may directly inhibit pubertal development of rat Leydig cells [46]. In humans, prenatal PFOS exposure may increase fetal steroid hormone production, although no association with cryptorchidism or hypospadias has been observed [47]. Anti-Müllerian hormone and INSL3 have been recently recognized as optimal markers of Sertoli and Leydig cells function, respectively, in particular during the first years of life [48]. However, modifications of their levels after EDs exposure have not been widely investigated in human studies. These reports may eventually provide additional insight on the pathophysiology of endocrine disruption on testis development.

## Effects of EDs on timing of puberty

The term puberty means a complex of psycho-neuro-endocrine changes that occur between the end of the childhood and the achievement of the complete sexual maturity. Usually, the normal length of puberty is about 5–6 years. Puberty usually begins later in males than in females, between 9.5 and 13.5 years old (on average 11.5 years old), and the first sign is the testes volume increase (>4 mL), followed by the pubarche within 6 months. After 12–18 months, the enlargement of penis is usually observed. All the clinical modifications that occur during the puberty are the consequence of the hypothalamus–pituitary–gonad axis activation, represented by the increase of the GnRH pulsatility and therefore of FSH, LH, and gonad steroids [49, 50]. The literature reports several studies investigating the relationship between potential endocrine-disrupting agents and the onset of puberty in boys and girls. Many cross-sectional and longitudinal human studies have evaluated the association between pubertal timing onset and prenatal or pubertal exposure to several chemical agents with plausible endocrine interference. Most studies were referred to girls, while few were committed for males’ puberty. Den Hond et al. [51] evaluated 80 boys who were exposed, during the pubertal period, to PCBs and dioxin. They found a negative association between the elevated serum PCBs exposure and pubertal stages, particularly the genital maturation and the pubic hair presentation. On the contrary, they did not find negative effects of dioxin. Saiyed et al. [52] reported an association between pubertal exposure to the pesticide endosulfan and low level of pubic hair, testis, and penis maturation, suggesting a delay in sexual maturation. A work published in 2008 involved 18 girls and 15 boys who were exposed to dioxin-contaminated breast milk. The authors observed a delayed breast development in females and a delayed age at first ejaculation in males [53]. Considering more recent works, Ferguson et al. longitudinally analyzed prenatal and infantile effects of phthalates and BPA on 118 boys (aged 8–14). Prenatal exposure was negatively associated to the adrenarche and pubarche onset (with high SHBG levels), whereas the infantile exposure caused low testosterone levels but no association with puberty was reported [54]. A similar work evaluated the impact of in utero phthalate and BPA exposure on the sexual pubertal maturation of 109 males. In particular, first- and second-trimester pregnancy exposure to DEHP was linked to increased peripubertal serum estradiol levels, whereas third-trimester exposure was associated to a delay on the onset of pubarche, with increased SHBG levels [55]. A longitudinal study conducted on 516 boys considered the effects of many organochlorine chemicals, lead (Pb) and non-dioxin-like PCBs. The authors evaluated EDs concentrations at the age of 8–9 years, and successively annual visits were carried out until the age of 18–19. The endure of blood EDs negatively influenced the growth during the puberty; in particular, dioxin-like compounds, organochlorine pesticides and the Pb delay puberty onset, whereas non-dioxin-like PCBs tend to advance puberty beginning [56]. These studies are summarized in Table 2.

**Table 2:** Main studies that investigated EDs effect on timing of male puberty

Authors [ref. No.]	Caseload	Analyzed EDs	Biological sample	Investigated time of exposure	Main findings
Den Hond et al. [51]	80 Belgian adolescents (mean age 17.3 ± 0.8 years), 40 from rural area, 40 from urban areas	PCBs (3 congeners) and dioxin-like compounds	Blood	Postnatal	Negative association between serum PCBs exposure and pubertal stages
Saiyed et al. [52]	70 Indian exposed boys vs. 47 controls (10–19 years of age)	Endosulfan	Blood	Postnatal	Negative association between aerial exposure to endosulfan and pubic hair, testis, and penis maturation
Leijs et al. [53]	15 Dutch boys (14–19 years of age)	Dioxin-like PCBs (3 congeners) and other dioxin-like compounds (PCDD/F)	Boys blood. PCDD/F also dosed in maternal milk	Prenatal/perinatal (PCDD/F also dosed in maternal milk) and postnatal	Correlation with delayed age at first ejaculation and PCBs values
Ferguson et al. [54]	118 Mexican boys (8–14 years of age)	Phthalates (9 metabolites) and BPA	Urine (maternal 3rd trimester or childhood)	Prenatal and postnatal	Prenatal exposure to most phthalates and BPA negatively associated to the adrenarche and pubarche onset. Childhood exposure not associated with puberty
Watkins et al. [55]	109 Mexican boys (8–14 years of age)	Phthalates (9 metabolites) and BPA	Maternal urine (maternal 1st, 2nd, 3rd trimester)	Prenatal	First-trimester pregnancy exposure to DEHP related to increased peripubertal serum estradiol levels. Third-trimester exposure to phthalates associated to a delay on the onset of pubarche
Sergeyev et al. [56]	516 Russian boys (annual visits from age 8–9 years to age 18–19 years)	Organochlorine chemicals	Blood (collected at age 8–9 years)	Postnatal	Negative influence on the growth during the puberty of persistent organochlorines and lead. Dioxin-like compounds, organochlorine pesticides and lead (Pb) delay puberty onset, while non-dioxin-like PCBs tend to move up the puberty beginning

*PCBs* polychlorinated biphenyls, *PCDD/F* polychlorinated dibenzo-p-dioxins and polychlorinated dibenzofurans, *BPA* bisphenol A, *DEHP* di-2-ethylhexyl phthalate

## Effects of EDs on anogenital distance

The AGD refers to the distance between the anus and the external genitalia and it is approximately twice the length in male compared to female newborns [57]. AGD is considered a broad biomarker capable of both retrospectively determine early life androgen disruption and predict late-life reproductive disorders in male offspring [57–59]. Prenatal androgen action determines reproductive organ size and AGD, and the action can be disrupted by EDs [60–62]. Further studies have identified a fetal “masculinization programming window” (MPW), a period from the second to the third month after conception when androgen action could be remarkably affected. The difference of AGD between male and female may be explained by divergent androgen secretions in this period [63]. This sexual dimorphism is apparent in rodents as well as humans [64, 65]. Reproductive parameters at birth and in adulthood appear to be influenced by androgen action in the MPW, similar to their correlation with AGD in human studies [58]. Therefore, a short male AGD is considered a marker of disrupted androgen action. In rodents, a short male AGD largely predicts adverse effect outcomes and it had been used for decades as a marker of impaired fetal androgen action [57].

### Animal studies

Compounds most frequently reported to affect male AGD are phthalate esters. Many have been tested in rats, with DBP and DEHP being the most prevalent. Fetal exposure to certain phthalates results in a short AGD in rat male offspring, without any significant effect on female AGD. It is, for the most part, a dose-dependent effect, where increasing dose levels result in progressively shorter AGD, as recently reported [66]. In mice, newborn males exposed to phthalates during the postnatal phase showed a significant short-term reduction of AGD as a possible result of prepubertal hormonal interference [67], although the exact mechanism is still unknown, given the embryonal determination of AGD.

Other substances with a clear antiandrogenic action can affect AGD in rat offspring. Prenatal exposure to high doses of certain AR antagonists, such as pesticide procymidone, vinclozolin, dichlorodiphenyltrichloroethane (DDT), and the non-steroidal prostate cancer drug flutamide, reduces male pup AGD up to 50% compared to controls [68–72]. After exposure to these compounds, the male offspring also displays an increased rate of nipple retention, genital malformations, and severely reduced male reproductive organ weights [57, 68, 73–75]. Fetal exposure to both the antimicrobial preservative butyl paraben [76, 77] and the industrial plasticizer BPA [78] has been shown to shorten male AGD around 7–16% in the male offspring, albeit there are studies reporting no effects on AGD for both butyl paraben [79] and bisphenol A [80–84]. PCB exposure in female rats during lactation resulted in reduced AGD in male progeny aged 60 days, even at the lowest dose tested, possibly through a reduction of circulating androgens [85]. Therefore, all these studies suggest that exogenous chemicals can affect AGD in pre- and peri-natally exposed animals.

### Human studies

Several epidemiological studies have investigated the effects of EDs on AGD, but the results have been controversial. Most studies focused on male infants, whereas less evidence is available to support a reduction in AGD following prenatal exposure to EDs even in puberty and transition periods. In fact, fetal exposure to different EDs has been frequently associated with a short AGD in newborn boys, in particular phthalates [86–90], but also PFAS [91], dioxins [92], BPA [93, 94], and DDT [95]. Notably, several studies have not found significant correlations between exposure levels and short AGD in boys, including some phthalates [96, 97], DDT [98], triclosan [99], PFAS [100], and various pesticides [101]. These discrepancies do not necessarily diminish the cause for concern, but rather highlight the challenges of obtaining evidence for causal relationships from human epidemiological studies [57]. Swan et al. [89] examined AGD and other genital measurements in relation to prenatal phthalate exposure in 134 newborns aged 2–36 months. The results showed that urinary concentrations of four phthalate metabolites and the summary score were inversely related to AGD. The conclusion supported the hypothesis that prenatal phthalate exposure at environmental levels may adversely affect male reproductive development in humans [89]. Successively, the same group confirmed these results in 2015 [90]; moreover, they observed the same association with reduced AGD when the daily exposures were substantially lower than current US Environmental Protection Agency (EPA) reference doses [102]. Suzuki et al. [88] examined the relationship between prenatal exposure to seven urinary phthalate ester metabolites and AGD in 111 newborns. The results showed the MEHP was negatively associated with AGD. In a Swedish cohort of 196 infants aged 21 months, Bornehag et al. [86] reported a reduced AGD in relation to phthalates concentration in mothers. In a more recent study, the authors investigated phthalates and BPA prenatal exposure in 198 male infants aged 6 months. Surprisingly, the results showed that both MBP and the molar sum of low molecular weight phthalates were positively associated with AGD, although no mechanism to explain this association was suggested [103].

Regarding puberty age, a study on 153 male children aged 0–17 years examined the effect of maternal and paternal exposure to BPA on AGD. Although the cohort included mainly children <5 years old, after correction for age the negative association between either paternal or maternal BPA exposure and AGD remained significant, although the effect was greater when considering mothers’ BPA levels [94]. Another study on young men aged 18–23 reported a reduced AGD associated with maternal exposure to pesticides during pregnancy [104], suggesting that the previously reported associations between EDs prenatal exposure and neonatal and perinatal AGD in male infants can be extended also to later ages. In the same way, these results were also recently confirmed for PFAS: in 212 young men aged 18–19 years, direct exposure to these chemicals resulted in a 10% reduction of AGD [91]. Although PFAS were measured directly in subjects, it could be considered a proxy of prenatal exposure, given the very long half-lives of PFAS [105] and the long-lasting pollution in the geographic area of the exposed subjects. Interestingly, the same study reported a significant negative association between PFAS exposure and another androgen-dependent parameter, penile length [91]. Human studies characteristics are summarized in Table 3.

**Table 3:** Main studies that investigated EDs effect on male anogenital distance (AGD)

Authors [ref. No.]	Caseload	Analyzed EDs	Biological sample	Investigated time of exposure	Main findings
Swan et al. [89]	134 US newborns (aged 2–36 months)	Phthalates (9 metabolites)	Maternal urine (1st trimester)	Prenatal	MEP, MBP, MBzP, and MiBP inversely related to anogenital index (AGD/weight)
Torres-Sanchez et al. [95]	37 Mexican infants (aged 3, 6, 12, 18 months)	DDT/DDE	Maternal blood during pregnancy	Prenatal	Significant reduction in AGD due to the prenatal DDE exposure, specifically during the first trimester of pregnancy (AGD reduction for doubling increase of maternal DDE)
Huang et al. [97]	33 Taiwanese newborns	Phthalates (5 metabolites)	Maternal urine and amniotic fluid	Prenatal	No correlation between in utero phthalates exposure and male AGD
Miao et al. [94]	153 Chinese boys (aged 0–17 years, 81% < 10 years old)	BPA	Parental urine. Personal air sample monitoring also carried out	Prenatal	Parental occupational exposure to BPA during pregnancy associated with shortened AGD (stronger for maternal exposure: AGD 8.11 mm shorter for boys with maternal exposure)
Suzuki et al. [88]	111 Japanese male newborns (aged 1–3 days)	Phthalates (7 metabolites)	Maternal urine (9th–40th week of gestation)	Prenatal	Significant inverse association between MEHP and reduced anogenital index (AGD/weight)
Bustamante-Montes et al. [87]	73 Mexican newborns (24 and 48 h after birth)	Phthalates (4 metabolites)	Maternal urine (3rd trimester)	Prenatal	Significant inverse association between total phthalate exposure and AGD
Vafeiadi et al. [92]	119 newborn and 239 infants (aged 16 months) from Greece and Spain	Dioxins and dioxin-like compounds	Maternal blood (after delivery)	Prenatal	Plasma dioxin-like activity negatively associated with AGD in male newborns (AGD reduced 0.44 mm per 10 pg toxic equivalent increase). Nonsignificant negative association in young boys
Bornehag et al. [86]	196 Swedish infants (aged 21 months)	Phthalates (10 metabolites)	Maternal urine (1st trimester)	Prenatal	Strongest and most significant inverse associations with DiNP metabolites, though with a small reduction in AGD
Swan et al. [90]	366 US newborns (aged 1–3 days)	Phthalates (11 metabolites)	Maternal urine (1st trimester)	Prenatal	Three metabolites of DEHP (MEHP, MEOHP, MEHHP) significantly and inversely associated with AGD
Jensen et al. [96]	245 Danish infants (aged 3 months)	Phthalates (12 metabolites)	Maternal urine around week 28 of gestation	Prenatal	No significant trends toward shorter AGD in boys with higher phthalates exposures
Bornman et al. [98]	344 infants from South Africa (visits at birth and 1 year)	DDT/DDE	Maternal blood	Prenatal	No associations between DDT/DDE congeners and AGD
Lassen et al. [99]	252 Danish infants (aged 2–7 months)	Triclorosan	Maternal urine during pregnancy (at approximately gestational week 28)	Prenatal	No significant association with a reduced AGD at 3 months of age in boys
Lind et al. [100]	316 Danish newborns (at age 3 months)	PFAS	Maternal blood during pregnancy (gestational week 5–12)	Prenatal	No association between PFAS and AGD in boys
Cremonese et al. [104]	Young Brazilian men, aged 18–23 years, from two different areas, rural (99 men) and urban (36 men)	Pesticides	No direct dosage, exposure evaluated through a questionnaire	Prenatal and postnatal	In rural men, AGD 9% larger (data not shown). Significant increase in AGD (5%) among men born to women who worked in agriculture during pregnancy
Di Nisio et al. [91]	Italian young boys, 171 controls (mean age 18.7 ± 1.0 years) and 212 exposed subjects (mean age 18.5 ± 0.8 years)	PFAS	Blood and seminal fluid (50 controls and 50 exposed males)	Prenatal/postnatal	Significant reduction in exposed group: AGD 0.4 mm shorter (4.5 vs. 4.1 mm); penile length 1.1 cm shorter (9.7 vs. 8.6 cm)
Mammadov et al. [93]	72 newborns aged 1–3 days from Cyprus	BPA	Umbilical cord specimen taken at birth	Prenatal	Significant association between high cord blood BPA levels and shortened AGD in male newborns (4.6 mm shorter between higher and lower exposed group)
Dalsager et al. [101]	420 Danish newborns (at age 3 months)	Pesticides	Maternal urine during pregnancy (at approximately gestational week 28)	Prenatal	No association between maternal urinary concentrations of pesticide metabolites and AGD in the offspring
Arbuckle et al. [103]	198 Canadian infants (aged 6 months)	Phthalates (11 metabolites), BPA, triclorosan	Maternal urine during pregnancy (1st trimester)	Prenatal	Long AGD measure positively associated with MBP and the molar sum of low molecular weight phthalates

*MEP* mono-ethyl phthalate, *MBP* mono-butyl phthalate, *MBzP* mono-benzyl phthalate, *MiBP* mono-isobutyl phthalate, *DDT* dichlorodiphenyltrichloroethane, *DDE* dichlorodiphenyldichloroethylene, *BPA* bisphenol A, *MEHP* monoethylhexyl phthalate, *DiNP* diisononyl phthalate, *MEOHP* mono(2-ethyl-5-oxohexyl) phthalate, *MEHHP* mono(2-ethyl-5-hydroxyhexyl) phthalate, *PFAS* per- and poly-fluoroalkyl substances

## Effects of EDs on penile length

Penile length is a parameter that positively correlates with postnatal androgen levels [74]. Several studies investigated this correlation, though in newborns. To date, there is only one study that evaluated penile length in pubertal boys in association with EDs exposure: in a cohort of 55 boys aged 11–14, maternal exposure to PCBs during pregnancy was associated with reduced penile length [106]. As previously mentioned, Di Nisio et al. observed similar results in young men aged 18–19, suggesting exposure to EDs can result also in reduced penis size in adolescence [91]. On the contrary, Leijs et al. reported no correlation in 15 young boys (14–19 years of age), exposed to PCBs and dioxin-like compounds, but results were not shown [53].

## Effects of EDs on testicular volume

Only few studies investigated the association between EDs and TV in pubertal or transition age. In most studies, TV has been evaluated only for staging puberty [54–56]. In addition, studies are extremely heterogeneous, investigating different EDs, considering different primary outcomes, and using different methods for testicular sizing. In fact, TV was evaluated using Prader orchidometer [51, 53, 107–109], ultrasound [91], both [110–112], or a digital caliper [104]. Moreover, TV may be affected by both in utero and adult exposure [113, 114], with no study investigating the one and the other at the same time. As said before, TV > 4 mL is considered the first pubertal sign, while a TV > 12 mL is considered normal at the end of puberty. Therefore, TV comparison was performed after adjustment for genital stage and in most studies was made according to tertiles or quartiles of exposure, without a control group. Mol et al. [110] observed no significant differences in mean TV, hormonal concentration, and Tanner stage after prenatal PCB exposure in a cohort of children examined at mid puberty. Grandjean et al. [112] evaluated from the same birth cohort 438 adolescent boys, at age 14 years, confirming the same results. On the contrary, Den Hond et al. evaluated postnatal PCB and dioxin-like compounds exposure in 80 boys coming from three different areas of Belgium. The authors reported differences in TV between the areas, even after adjustment for genital stage. However, TV did not correlate with any of the investigated biomarkers of exposure, suggesting it may be a consequence of maternal exposure or other not explored factors [51]. Leijs et al. reported the same no association, but results were not shown [53]. Different results were observed by Cremonese et al., though they used a questionnaire to investigate occupational exposure to pesticides in young Brazilian men. Testicles were larger in rural men, in men using pesticides for more than 1 year, and also among men born to women who worked in agriculture during pregnancy [104]. This is the only study reporting an increase in TV. Authors suggested two possible mechanisms: fetal life exposure with prevalent androgen effect or adult exposure with inflammation of the testicles. Concerning other EDs, Vested et al. [107] recruited 169 young male, aged 19–21 years, whose mothers’ blood had been previously collected during pregnancy. No correlation between PFOA, PFOS, and self-measured TV was observed [107]. Same results were recorded by Joensen et al., who investigated PFAS postnatal exposure in 247 randomly selected healthy young Danish men [111]. On the contrary, Di Nisio et al. reported smaller mean TV in exposed PFAS group (mean age 18.5 ± 0.8 years). Interestingly, both serum and semen PFOA, but not PFOS, were associated with reduced TV [91]. Regarding phthalates, Axelsson et al. investigated prenatal exposure in 112 young Swedish men, aged 17–20 years. Maternal blood samples were recovered in a biobank. The only significant result was that men in the highest tertile of prenatal mono(carboxy-isooctyl) phthalate exposure had smaller TV than men in the lowest [108]. On the contrary, Durmaz et al. found plasma levels of DEHP and MEHP significantly higher in 40 newly diagnosed pubertal gynecomastia cases, aged 11–15 years. However, there was no significant association between their concentration and TV [109]. Studies characteristics are summarized in Table 4.

**Table 4:** Studies that investigated EDs effect on testicular volume (TV) in pubertal/transition age boys

Authors [ref. No.]	Caseload	Analyzed EDs	Biological sample	Investigated time of exposure	Main findings
Mol et al. [110]	176 children born in 1986–1987 in Faroe Islands, examined approximately at mid puberty (mean age 13 years and 9 months)	PCBs (3 congeners)	Umbilical cord specimen taken at birth	Prenatal	No significant difference in mean TV
Den Hond et al. [51]	80 Belgian adolescents (mean age 17.3 ± 0.8 years), 40 from rural area, 40 from urban areas	PCBs (3 congeners) and dioxin-like compounds	Blood	Postnatal	Significant difference in TV between the areas, even after adjustment for genital stage (left plus right TV 46.8 mL in rural area vs. 44.1 and 41.4 mL in the two polluted area). TV did not correlate with any of the biomarkers of exposure investigated.
Leijs et al. [53]	15 Dutch boys (14–18.7 years of age)	PCBs (3 congeners) and dioxin-like compounds (PCDD/F)	Blood; PCDD/F also dosed in mothers’ milk	Prenatal/Perinatal (PCDD/F also dosed in mothers’ milk) and postnatal	No association with exposure and TV (results not shown)
Durmaz et al. [109]	40 newly diagnosed pubertal gynecomastia cases, aged 11–15 years (mean age 13.2 ± 0.9 years) vs. 21 controls	Phthalates (2 metabolites)	Blood	Postnatal	Plasma levels of DEHP and MEHP significantly higher in the pubertal gynecomastia group; no significant association between their concentration and TV
Grandjean et al. [112]	438 14-year-old boys born in 1986–1987 in Faroe Islands	PCBs (3 congeners)	Umbilical cord specimen taken at birth	Prenatal	Nonsignificant inverse association with TV
Vested et al. [107]	169 young male, aged 19–21 years, from a Danish pregnancy cohort established in 1988–1989	PFAS	Maternal blood (pregnancy week 30)	Prenatal	No significant differences in TV between all groups of exposure
Joensen et al. [111]	247 Danish men (mean age 19.6 ± 1.4 years)	PFAS	Blood	Postnatal	No association between TV and serum PFAS levels (results not shown)
Axelsson et al. [108]	112 young Swedish men, aged 17–20 years (mean age 18.3 ± 0.41 years).	Phthalates (6 metabolites)	Maternal blood (69% of samples between 8 and 14 gestational weeks)	Prenatal	Significant smaller TV in the highest tertile of prenatal MCiOP exposure than men in the lowest (left plus right 41 vs. 45 mL)
Cremonese et al. [104]	Young Brazilian men, aged 18–23 years, from two different areas, rural (99 men) and urban (36 men).	Pesticides	No direct dosage, exposure evaluated through a questionnaire	Prenatal and postnatal	In rural men, testicles 31% larger (mean TV 26.3 vs. 19.2 mL), also increased in men using pesticides for more than 1 year. A significant increase in TV (16%) observed among men born to women who worked in agriculture during pregnancy
Di Nisio et al. [91]	Italian young boys, 171 controls (mean age 18.7 ± 1.0 years) and 212 exposed subjects (mean age 18.5 ± 0.8 years)	PFAS	Blood and seminal fluid (50 controls and 50 exposed males)	Prenatal/postnatal	Smaller mean TV in exposed group (14.7 vs. 16.1 mL). Both serum and semen PFOA, but not PFOS, associated with reduced TV

*PCBs* polychlorinated biphenyls, *PCDD/F* polychlorinated dibenzo-p-dioxins and polychlorinated dibenzofurans, *DEHP* di-2-ethylhexyl phthalate, *MEHP* mono-ethyl-hexyl phthalate, *PFAS* per- and poly-fluoroalkyl substances, *MCiOP* mono-(carboxy-iso-octyl) phthalate, *PFOA* perfluorooctanoic acid, *PFOS* perfluorooctanesulfonic acid

## Effects of EDs on testicular cancer

Few studies investigated a possible correlation between EDs exposure and TC. None of these was performed specifically in adolescents or in transition age. Some papers did not report the age of the studied group or all included patients were older than transition age [115–119]. However, TC is the most common tumor diagnosed in men aged 14–44 years [120]. Most studies evaluated a possible correlation between TC and PCBs. Indeed, the International Agency for Research on Cancer (IARC) rendered PCBs as definite carcinogens in humans [121], while according to the US EPA PCBs cause cancer in animals and are probable human carcinogens (http://www.epa.gov/epawaste/hazard/tsd/pcbs/pubs/effects.htm). However, results regarding TC, as well as other neoplasms, are limited and often discordant. The only study taking into consideration a subgroup of adolescents was performed by Koifman et al. An epidemiological study was carried out by comparing the total amount of pesticides sales in 1985 and health data traced in a National database. A non-statistically significant correlation was observed for TC hospitalization in both age groups (0–14, 15–49 years old) [122]. However, authors did not quantify either the exact amount of pesticides used or the exact area where they were used. In a similar registry-based study, Le Cornet et al. showed no evidence of an association between parental exposure to pesticides and TC [123]. In a cohort of US soldiers, McGlynn et al. reported an increased TC risk in patients with higher plasma levels of DDE and chlordane components [124]. Surprisingly, postnatal PCBs exposure was associated with decreased TC risk in the same cohort [125]. At the same way, Biggs et al. evaluated 246 TC patients after the first course of cancer treatment; however, only 33 were aged 18–24 years (13,4%). TC risk was similar across groups and no trend with increasing serum pesticide levels was observed [126]. Different results were reported by Paoli et al., who observed a statistically significant increase in TC risk in cases with detectable values of total PCB [127]. On the contrary, Hardell et al. investigated both prenatal and postnatal PCB exposure. In patients, only the concentration of *cis*-nonachlordane was significantly increased, whereas their mothers showed significantly increased concentrations of the sum of PCBs, hexachlorobenzene, *trans*- and *cis*-nonachlordane, and the sum of chlordanes [128, 129]. Studies characteristics are summarized in Table 5.

**Table 5:** Studies that investigated EDs effect on testicular cancer (TC) in pubertal/transition age boys

Authors [ref. No.]	Caseload	Analyzed EDs	Biological sample	Investigated time of exposure	Main findings
Koifman et al. [122]	Brazilian men, divided in two age-specific groups: 0–14 and 15–49 years old (number of patients not shown)	Pesticides	No direct dosage. Pesticides sales in 1985 compared with TC hospitalization in 1995–1997 and TC chemotherapy in 1999–2000	Prenatal	No significant correlation for TC hospitalization in both age groups
Hardell et al. [128, 129]	58 Swedish TC patients aged 18–45 vs. 61 controls. 44 cases’ mothers vs. 45 controls’ mothers	PCBs (37 congeners), HCB, DDE, and chlordanes (6 congeners)	Blood	Prenatal and postnatal	In cases significantly increased concentration of *cis*-nonachlordane; significantly increased concentrations of the sum of PCBs, HCB, *trans*- and *cis*-nonachlordane, and the sum of chlordanes in cases’ mothers
Biggs et al. [126]	272 US TC patients aged 18–44 years after the first course of cancer treatment (13.4% aged 18–24 years) vs. 630 controls	PCBs (36 congeners), DDE, HCB, and other pesticides	Blood	Postnatal	Similar TC risk across categories of serum PCB concentration, no trend with increasing serum pesticide levels
McGlynn et al. [124]	US military personnel. 915 controls and 739 cases (8.7% 18–20 years old, 32.5% 21–25 years old)	DDE, HCB, and other organochlorine compounds	Blood (previously stored)	Postnatal	TC risk associated with higher plasma levels of DDE and of two chlordane components, *cis*-nonachlor and *trans*-nonachlor
McGlynn et al. [125]	US military personnel. 913 controls and 736 cases (8.7% 18–20 years old, 32.3% 21–25 years old)	PCBs (15 congeners)	Blood (previously stored)	Postnatal	Statistically significant decreased risk of TC in association with eight PCBs and with overall sum.
Le Cornet et al. [123]	9569 North European men aged 14–49 years at TC diagnosis (85% 20–40 years old) vs. 32,028 controls.	Pesticide, fungicide, herbicide, and insecticide	No direct dosage. Parental occupation collected and converted into a pesticide exposure index	Prenatal	No association between parental exposure and TC
Paoli et al. [127].	125 Italian TC patients (aged 29.6 ± 5.9 years) vs. 103 controls	PCBs (9 congeners), HCB	Blood	Postnatal	Statistically significant increase in TC risk in cases with detectable values of total PCB

*PCBs* polychlorinated biphenyls, *HCB* hexachlorobenzene, *DDE* dichlorodiphenyldichloroethylene

## Limitations

Several studies have focused on the effects of EDs, especially on testis development; however, they used different animal models, different EDs doses, and different methodologies, with results not easily comparable among them. In humans, there are only few original papers on the effects of EDs on puberty in males, as the majority focus on female puberty. This aspect could find an explanation in the fact that females’ puberty is more easily detectable, since menarche represents an undisputed sign of sexual maturation. Given the lack of homogeneity across the different studies, because of the wide spectrum of EDs and different exposure sources, age of subjects, and analytical measurements, it is difficult to infer a comprehensive conclusion on the effect of EDs on puberty and transition period. Moreover, we should consider the lack of longitudinal studies in the literature, and in most cases the quantification of EDs levels was performed only in mothers during pregnancy or only in children after birth, thus not providing a direct assessment of mother–fetus exposure levels. In other cases, the analytical measurement of chemicals is missing and it is only presumed by occupational exposure. In addition, humans are exposed to several chemicals, which could affect male health in a dose-additive manner. In fact, testicular toxicity was reported in rats exposed to a mixture of phthalates, though dosage of each was below the adverse effect threshold [130]. Therefore, bioaccumulation in adipose tissue of several EDs may be responsible of a “cocktail effect,” a possible sum of effects with unknown outcomes that may unexpectedly appear after years of exposure to low dosages [131]. Moreover, as said before, recent data suggest that EDs detrimental effects may even be inherited by future generations [132]. All these factors may partially contribute to the heterogeneity of results reported in literature. In the same way, lifestyle factors in young adults should not be neglected. An increasing trend of health risk behaviors, such as smoking, use of illegal drugs and alcohol, has been recently reported in adolescents. All these behaviors have been associated with andrological disorders and could impair testicular development [133].

## Conclusions

**Fig. 1:**
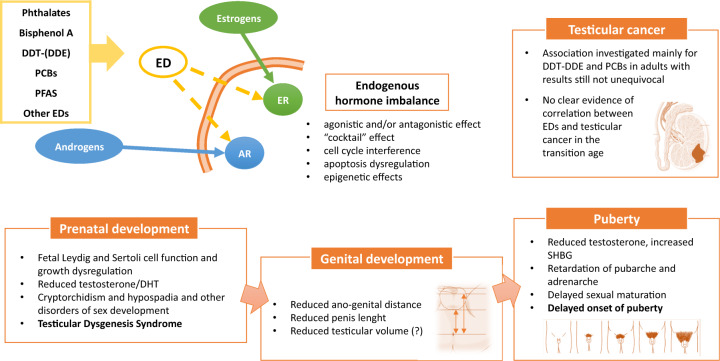
Endocrine disruptors mechanisms of endogenous hormone imbalance and the adverse consequences on male puberty, genital development, and andrological health

Current evidence does not clarify the impact of EDs on human male reproductive health. In animal models, severe harmful effects were observed. However, human studies have shown controversial results. This discrepancy may be due to several factors, as said above. Despite the lack of consistency in the results, overall conclusion points toward a positive association between exposure to EDs and reproductive system damage. Among the studies based on the consequences of EDs exposure on males’ puberty, the main findings concern on delayed puberty, probably associated to the xeno-estrogens effects of PCBs, polychlorinated dibenzofurans, and endosulfan. In the same way, by using AGD as a proxy of fetal exposure to endocrine-disrupting chemicals, most studies agree on an antiandrogenic effect of different classes of EDs (phthalates, BPA, pesticides, PFAS), which results in reduced AGD after birth, as measured from newborns until transition period. On the contrary, the correlation between EDs and TV, as well as that with testicular cancer, is more uncertain. EDs mechanisms of damage and the adverse consequences on male puberty and andrological health are summarized in Fig. 1. Although the observed effects may be subtle on an individual level, the biological link between them (i.e., TDS: decreased androgen levels contributing to cryptorchidism, reduced penile length, reduced TV) should raise concern about the effects of EDs at population levels in young men. Further long-term studies performed on a wide number of subjects are necessary in order to identify damaging compounds, clarify sources of exposure, and replace them with harmless substances.
